# Case report: Characterization of a patient with novel biallelic variants in *GTF3C3* and comprehensive review of previously reported cases

**DOI:** 10.3389/fgene.2026.1860633

**Published:** 2026-06-30

**Authors:** Lucía López-López, Beatriz González Giráldez, Yolanda Benítez, Belén Benavides, Carmen Ayuso, Pablo Mínguez, Fiona Blanco-Kelly, Berta Almoguera

**Affiliations:** 1 Department of Genetics & Genomics, Fundación Jiménez Díaz University Hospital, Health Research Institute-Fundación Jiménez Díaz University Hospital, Universidad Autónoma de Madrid (IIS-FJD, UAM), Madrid, Spain; 2 Center for Biomedical Network Research on Rare Diseases (CIBERER), Instituto de Salud Carlos III, Madrid, Spain; 3 Bioinformatics Unit, Health Research Institute-Fundación Jiménez Díaz University Hospital, Universidad Autónoma de Madrid (IIS-FJD, UAM), Madrid, Spain

**Keywords:** epilepsy, genetics, *GTF3C3*, intellectual disability, neurodevelopmental disorder

## Abstract

Biallelic variants in *GTF3C3* have been recently associated with an autosomal recessive form of syndromic intellectual disability, with only 16 patients reported to date. Using whole exome sequencing, we identified previously unreported biallelic *GTF3C3* variants in a new patient, who also represents the oldest reported individual so far. We detail the genotype and phenotype of this case and provide a comprehensive review of all published patients to better define the clinical features and their frequency. The most consistent manifestations across the cohort include global developmental delay, intellectual disability, epilepsy, central nervous system malformations, and additional neurological abnormalities. This case, together with the literature review, further delineates the phenotype associated with *GTF3C3*.

## 1 Introduction

*GTF3C3* (OMIM 604888), located in chromosome 2, encodes a subunit of the DNA-binding transcription factor IIIC (TFIIIC) complex (Seifert-Davila et al., 2023). This complex plays a crucial role in RNA-mediated transcription by recruiting RNA polymerase III and participates in chromatin remodeling, through its histone acetylation activity (Winter et al., 2000; Sinn et al., 1995), thereby contributing to genome organization and gene expression control.

Very recently, two independent studies have established biallelic variants in *GTF3C3* as the cause of an autosomal recessive form of syndromic intellectual disability (ID) (MIM 621201) in 16 patients (De Hayr et al., 2025; Abdel-Hamid et al., 2025). All 16 reported individuals present with a phenotype characterized by neurocognitive impairment, frequently accompanied by microcephaly, central nervous system malformations, dysmorphic features, and seizures.

In this study, we describe a previously unreported patient carrying novel biallelic variants in *GTF3C3* who represents the oldest reported individual to date with this condition. In addition, we also present a comprehensive review of the phenotype reported in previously described cohorts delineating the clinical characteristics of the syndrome and their frequency.

## 2 Materials and methods

This study was approved by the Ethics Committee of Fundación Jiménez Díaz University Hospital (FJD-UH; PIC202-23_FJD) and carried out in accordance with the principles of the Declaration of Helsinki and all applicable institutional regulations. Written informed consent was obtained from the patient.

Genomic DNA was extracted from peripheral blood samples collected in EDTA tubes from the patient and her parents. Clinical Exome sequencing was done using the Clinical Exome Solution (CES) v3 from SOPHiA Genetics and sequenced on a NextSeq2000 instrument (Illumina) which includes the coding region of 4,500–5,000 genes included in human genetic conditions. Whole-exome sequencing (WES) was performed using the Human Core Exome library (Twist Bioscience), sequenced on a NovaSeq X system (Illumina, San Diego, CA, United States) and analyzed with the FJD-Pipeline (https://github.com/TBLabFJD/PARROT-FJD). Variants were classified following the guidelines of the American College of Medical Genetics and Genomics (ACMG) (Richards et al., 2015). The identified variants were confirmed by Sanger sequencing and segregated in the parents.

## 3 Case description

Our patient is a 33-year-old Spanish female, born to non-consanguineous parents, with no relevant family history. Pregnancy was unremarkable, and she was born at 37 weeks of gestation via spontaneous vaginal delivery. Her birth weight was 2500gr (∼p10) and she presented with transient neonatal hypotonia. Psychomotor development was delayed from early infancy; she achieved independent walking at 2 years and 9 months, reported a history of frequent falls, and required academic support throughout her education due to learning difficulties and borderline intellectual functioning (low-average IQ). In the context of her developmental delay, brain MRI performed during childhood revealed cerebellar hypoplasia.

At 22 years of age, she experienced her first generalized tonic-clonic seizure during wakefulness. Prolonged EEG revealed mild-to-moderate diffuse background slowing, with additional focal bitemporal slowing and interictal generalized epileptiform discharges. Frequent generalized tonic-clonic seizures persisted for years, both during wakefulness and sleep, and were finally controlled at age 27 with low doses of clobazam. At that moment, brain MRI confirmed marked cerebellar hypoplasia involving both the vermis and hemispheres. Mild supratentorial cortico-subcortical atrophy was also noted. Follow-up brain MRIs have shown radiological stability since then.

On neurological examination, she exhibited gait ataxia with instability and difficulty coordinating head movements while walking. Additional findings included dysarthria, reduced verbal fluency, mild intentional tremor and dysdiadochokinesia.

She had mild facial dysmorphic features including long eyelashes, hypertelorism, a broad nasal bridge with a full nasal tip, tented upper lip and a full lower lip. Dental anomalies were also noted, including macrodontia of the permanent maxillary central incisors and diastema.

Chromosomal analysis showed a normal female karyotype (46, XX) and the CGH array study as well as the CES analysis, which includes 759 genes associated with neurodevelopmental disorders (NDDs), were negative. Due to the non-informative results from the CES, the patient was subjected to WES within our cohort study (López-López et al., 2026), where the analysis showed two novel variants in the *GTF3C3* gene (NM_012086.5): c.1708C>T (p.Arg570*) and c.2498 A>G (p.Tyr833Cys), that were further confirmed by Sanger sequencing to be inherited from the father and the mother, respectively. The c.1708C>T (p.Arg570*) variant is classified as likely pathogenic (class 4) according to the ACMG guidelines, criteria PM2 and PVS1. The c.2498 A>G (p.Tyr833Cys) variant is classified as VUS (class 3) based on the ACMG criteria including PM2, PP3 and PM3.

## 4 Review of the genotype and phenotype associated with *GTF3C3*

**FIGURE 1:**
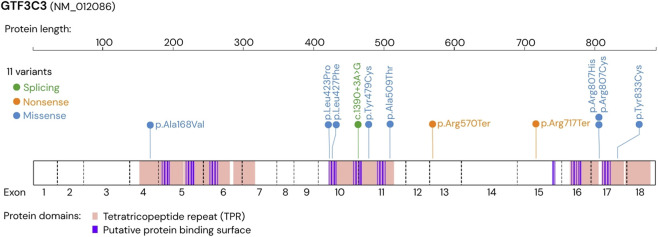
Schematic representation of the GTF3C3 protein showing the location of reported variants. Functional domains are indicated, together with the positions of variants identified in the present study and previously reported cases. Variant types are colored according to their molecular consequence.

The genotype and clinical features of all previously reported patients, along with the individual described in this study, are summarized in Table 1. A total of 11 pathogenic or likely pathogenic variants in *GTF3C3* have been reported to date, including eight missense, one splicing, and two nonsense variants. Among these, only one missense variant has been found recurrently, the missense variant c.503C>T (p.Ala168Val), identified in seven patients from four unrelated families. In our patient, we identified two novel biallelic variants: one missense and one nonsense. All variants, including the ones found in our patient, have a CADD higher than 25 or a SpliceAI higher than 0.6 and are present in the GnomAD database (Gudmundsson et al., 2021) at a frequency lower than 0.0001. Additionally, the majority of these variants (8 out of 11, 72.7%) are located within the tetratricopeptide repeat (TPR) domains of the protein (Figure 1).

**TABLE 1:** Genotypic and phenotypic features of all reported patients with *GTF3C3* variants, including the individual described in this study. Data from previously published cases were extracted from Reuter et al. (2017); Papuc et al. (2019); Anazi et al. (2017); De Hayr et al. (2025); Abdel-Hamid et al. (2025).

	P1 This study	P2 (De Hayr et al., 2025; Papuc et al., 2019)	P3 (De Hayr et al., 2025) (sibling of P4)	P4 (De Hayr et al., 2025) (sibling of P3)	P5 (De Hayr et al., 2025) (cousin of P3/P4)	P6 (De Hayr et al., 2025)	P7 (De Hayr et al., 2025) (sibling of P8)	P8 (De Hayr et al., 2025) (sibling of P7)
Age	33 y	24 y	6 y	17 y	2 y	3 y	15 y	9 y
Sex	Female	Female	Female	Male	Female	Male	Male	Male
Ethnicity	Spanish	Swiss	Lebanese	Lebanese	Lebanese	Lebanese	Egyptian	Egyptian
Consanguinity	-	-	+	+	+	+	+	+
Variants	c.2498 A>G (p.Tyr833Cys)/c.1708C>T (p.Arg570Ter)	c.503C>T (p.Ala168Val)/c.2419C>T (p.Arg807Cys)	c.503C>T (p.Ala168Val)	c.503C>T (p.Ala168Val)	c.503C>T (p.Ala168Val)	c.503C>T (p.Ala168Val)	c.503C>T (p.Ala168Val)	c.503C>T (p.Ala168Val)
Genotype	Compound heterozygous	Compound heterozygous	Homozygous	Homozygous	Homozygous	Homozygous	Homozygous	Homozygous
Variant type	Missense/Nonsense	Missense	Missense	Missense	Missense	Missense	Missense	Missense
CADD	28.3/37	36/33	36	36	36	36	36	36
TPR domain	11/-	1/-	1	1	1	1	1	1
NDD	Moderate ID and mild psychomotor retardation	Severe ID	Severe ID	Severe ID	Moderate ID	Severe ID	Moderate ID, mild ASD	Moderate ID, ild ASD
Seizures/age of onset	+ / 22 y	+ / 4 y	+ / NA	+ / NA	+ / NA	+ / 6 y	+ / 2 y	+ / 3 y
Semiology	GTC, myoclonic	Myoclonic, GTC, focal impaired awareness, reflex seizures	Absence seizures	Clonic seizures	NA	Staring with tonic posturing of arms	Myoclonic, versive seizures and infrequent generalized tonic	Generalized tonic, myoclonic
Current frequency	Free since age 27 y	1–3 focal impaired awareness seizures per month, GTC seizures rare	Occasional	NA	NA	Several times a week before starting treatment	Once per week	Daily (5–8 times)
Antiepileptic treatment	Valproate and clobazam	Valproate and clobazam	NA	Valproate and midazolam	Valproate and midazolam	Valproate	Valproate, levetiracetam	Valproate
Brain imaging	Vermian cerebellar hypoplasia, atrophy of cerebellar hemispheres	CC hypoplasia at age 8m; progressive, severe cerebral and marked CBA with simplified gyral pattern and hypoplastic frontal lobes bilaterally at age 15 y	CBA, cerebellar hypoplasia	Progressive cerebellar volume loss, posterior fossa arachnoid cyst and evidence of cortical dysplasia in the insular regions bilaterally	Progressive cerebellar volume loss, posterior fossa arachnoid cyst and evidence of cortical dysplasia in the insular regions bilaterally	MRI pending (NA)	-	CBA, thin CC/12.7 y
Microcephaly	+	+ (secondary)	-	-	-	+	-	-
Neurological features	Upper limb intention tremors, ataxic gait, dysarthria	Limb spasticity, spastic dyskinetic tetraparesis, abnormal conjugate eye movement and impaired smooth pursuit, choreoathetotic movement disorder at age 2m–6 m	Dystonia/spasticity, generalized rigidity	Dystonia/spasticity, hyperreflexia, clonus	Hyperreflexia, dystonia, spasticity and clonus	Foot clonus and lower limb tremors, diagnosis of hypotonic cerebral palsy	Hand tremors, ataxia	Hand tremors, ataxia
Muscle tone abnormalities	Transient hypotonia	-	-	-	-	Limb hypertonia, central hypotonia	Hypotonia	Hypotonia
Dysmorphisms	Long eyelashes, hypertelorism, broad nasal bridge, full nasal tip, tented upper lip, full lower lip, macrodontia of permanent maxillary central incisor, diastema	Abnormality of the hairline, widow’s peak, broad eyebrow, high, narrow palate, macrodontia of permanent maxillary central incisor, diastema	Curved eyebrow, broad nasal tip, pierre robin sequence (cleft palate and micrognathia)	Full brow, broad nasal tip, pierre robin sequence (cleft palate and micrognathia), thickened features/full lips	Curved eyebrows, almond eyes, broad nasal tip, long philtrum, large ears	High forehead, mild esotropia, bulbous nasal tip	High forehead, bushy thick eyebrows, straight palpebral fissures with almond shape eyes, broad nose and triangular, bulbous tip of nose, flat philtrum, straight lips, broad chin, low set ears	High forehead, bushy thick eyebrows, straight palpebral fissures with almond shape eyes, broad nose and triangular, flat philtrum, straight lips with v shaped upper one, broad chin, low set ears
Gastrointestinal anomalies	Disphagy	+	+	-	-	-	-	-
Other anomalies	Miopy and astigmatism	Short distal phalanx of finger, overlapping toe, short foot bilateral, scoliosis, delayed puberty	Pes cavus		-	-	-	
ClinVar accession IDs	VCV004819552.1 VCV004819553.1	VCV003899893.1 VCV003899892.1	VCV003899893.1	VCV003899893.1	VCV003899893.1	VCV003899893.1	VCV003899893.1	VCV003899893.1

P9 (Abdel-Hamid et al., 2025)	P10 (De Hayr et al., 2025)	P11 (Abdel-Hamid et al., 2025) (sibling of P12)	P12 (Abdel-Hamid et al., 2025) (sibling of P11)	P13 (De Hayr et al., 2025; Anazi et al., 2017) (sibling of P14)	P14 (De Hayr et al., 2025) (sibling of P13)	P15 (De Hayr et al., 2025; Reuter et al., 2017) (sibling of P16)	P16 (De Hayr et al., 2025; Reuter et al., 2017) (sibling of P15)	P17 (Abdel-Hamid et al., 2025)
17 y	7 y	2 years 8 m	1 year 9 m	5y9m	NA	13 y	22 y	2 y
Male	Male	Female	Female	Female	Male	Female	Female	Male
Iceland	Dutch	Egyptian	Egyptian	Saudi arabian	Saudi arabian	Turkish	Turkish	Brazil
-	-	+	+	+	+	+	+	-
c.514T>G (p.Cys172GLy)	c.1268T>C (p.Leu423Pro)/c.2420G>A (p.Arg807His)	c.1279G>T (p.Val427Phe)	c.1279G>T (p.Val427Phe)	c.1390 + 3 A>G	c.1390 + 3 A>G	c.1436 A>G (p.Tyr479Cys)	c.1436 A>G (p.Tyr479Cys)	c.2149C>T (p.Arg717Ter) c.1525G>A (p.Ala509Thr)
Homozygous	Compound heterozygous	Homozygous	Homozygous	Homozygous	Homozygous	Homozygous	Homozygous	Compound heterozygous
Missense	Missense/Missense	Missense	Missense	Splicing	Splicing	Missense	Missense	Nonsense/Missense
29.2	29.9/28.6	27.8	27.8	12.73	12.73	27.8	27.8	37/29.5
1	7/-	7	7	7–8	7–8	8	8	-/9
Moderate ID	Mild ID, clinical suspicion of ADHD	Severe ID, autistic features	Severe ID, autistic features	Severe ID	Severe ID	Moderate ID	Mild ID	ID
-	+ / 11 m	+ / 1 y	-	-	+/1 y	+/16 m	+/NA	+/1 y
-	No definitive seizures but suspected seizures in past. Anti-epileptic drugs until 4 y	GTC	-	NA	Tonic	Two febrile seizures with EEG abnormalities, a few absences	Absences	Tonic seizures
-	-	NA	-	NA	Only once	Free since age 6 years 6 m	No seizures, but still EEG anomalies at 22 y	NA
-	Prior valproate, carbamazepine	NA	-	NA	Off treatment at the age of 3 y	No medication since age 10 y	Ethosuximide	NA
Diffuse simplified gyral pattern with pontocerebellar hypoplasia	MRI cerebrum: Megacysterna magna	Mild diffuse brain volume loss with slightly thin CC and predominant CBA. Ex-vacuo DLV more on left side	Mildly DLV, thin CC, mild CBA	MRI at 7 m prominent cortical sulci, dandy walker variant, aplasia/hypoplasia of the CC	Diffuse brain atrophy with thinning of CC	MRI in Turkey at age 2 years reported as abnormal, no details available	-	Slightly short CC, with relative hypoplasia of the frontal lobes, and diffuse CBA
+	+	+	+	+	+	-	-	+
NA	Lower limb spasticity, myoclonus	Unsteadiness	Unsteadiness, drooling	Head ataxia	Spastic quadriplegia	Ataxic gait, balance problems, sporadically dystonic movement of the hands	NA	NA
Mild hypotonia	-	Hypotonia	Hypotonia	Hypotonia	Axial hypotonia	-	-	Hypertonia
Long face, bifrontal narrowing, infraorbital crease, smooth philtrum, full lower lip	High forehead, long eye lashes, blue eyes, down slanting palpebral fissures, small nose, full lips, wide palate, broad jaw	Bifrontal narrowing, long eyelashes, upslanting palpebral fissure, hypertelorism, full cheeks, full nasal tip, tented upper lip, full lower lip	Round face, frontal prominence, bifrontal narrowing, long eyelashes, hypertelorism, short nose, broad nasal bridge, full nasal tip, full cheeks, tented upper lip, full lower lip	Facial asymmetry, bilateral temporal narrowing, epicanthal folds, upslanting palpebral fissures, bulbous nose and full cheeks	Upslanting palpebral fissure, bulbous nose and full cheeks epicanthal folds	Low frontal hairline, synophrys, broad nasal bridge, overbite, hypertrophic buccal mucosa, single transverse palmar crease right hand, slim fingers, slim feet with hippocratic nails, dry skin with hyperpigmentation on right abdomen, hypertrichosis	Low frontal hairline, thick eyebrows, broad nasal back, wide palpebral fissures, dry skin	Low anterior hairline, bifrontal narrowing, hypertrichosis over forehead, synophrys, long eyelashes, hypertelorism, upslanting palpebral fissures, epicanthus, full cheeks, short nose, full nasal tip, tented upper lip, full lower lip
Chronic constipation	Constipation	-	-	+	+	+	-	Nasoenteral tube
Retinopathy, bilateral syndactyly of toes II and III, genu valgus, pes valgus				-	-	Hearing loss, postaxial hexadactyly, radioulnar synostosis	Hearing los, postaxial hexadactyly, radioulnar synostosis	Nystagmus, hearing loss, thrombocytopenia, anemia
VCV003899897.1	VCV004819554.1 VCV003899894.1	VCV003899896.1	VCV003899896.1	VCV003233390.3	VCV003233390.3	VCV003899890.1	VCV003899890.1	VCV003899898.1 VCV003899899.1

Abbreviations, symbols and notation: ADHD: attention deficit hyperactivity disorder; ASD: autism spectrum disorder; CBA: cerebellar atrophy; CC: corpus callosum; DLV: dilated lateral ventricles; GTC: generalized tonic-clonic; m: months; MRI: magnetic resonance imaging; NA: not available; y: years; “+“: positive; “-“: negative.

All 17 patients exhibited developmental delay and mild to severe ID, accompanied by facial dysmorphisms, most notably a narrow forehead (11/17, 64.71%) and a full nasal tip (12/17, 70.59%), as well as microcephaly (10/17, 58.82%). Brain MRI, available for 16 patients, revealed abnormalities in 14 (87.5%), primarily affecting the cerebellum (10/16, 62.5%) and/or the corpus callosum (7/16, 43.75%). Seizures were present in the majority of cases (14/17, 82.35%), with varying types reported, including tonic, clonic, and absence seizures. Neurological signs were noted in 14 patients (82.35%) frequently including hand tremors or limb spasticity, and 10 patients (58.82%) showed abnormalities in muscle tone. Gastrointestinal manifestations were also relatively frequent, observed in nine out of 17 individuals (52.94%). In addition to the core features, less frequent but clinically significant manifestations included hearing loss in three individuals (3/17, 17.64%) and retinopathy in one (1/17, 5.88%).

## 5 Discussion

*GTF3C3* has only recently been implicated in an autosomal recessive syndromic NDD, with the first cases reported in 2025. It encodes a subunit of the TFIIIC, which plays a key role in RNA polymerase III–mediated transcription, essential for normal neurodevelopment. To date, only 16 individuals with biallelic variants in *GTF3C3* have been described (De Hayr et al., 2025; Abdel-Hamid et al., 2025), underscoring the rarity of the condition and the limited knowledge about its full phenotypic spectrum. Here, we describe a new patient carrying novel biallelic variants in *GTF3C3*, who represents the oldest reported case to date.

In our patient, we identified two novel biallelic variants, one missense and one nonsense. The missense variant is located within a TPR domain, consistent with the majority of previously reported missense variants. These domains are key structural motifs involved in protein-protein interactions and multiprotein complexes formation (Zeytuni and Zarivach, 2012). The clustering of variants within TPRs suggests their likely functional relevance in disease pathogenesis. In contrast, the nonsense variant lies outside these domains, as well as the other reported nonsense variant. However, they introduce a premature termination codon resulting in protein truncation that aligns with a loss of function mechanism, which is the main pathogenic mechanism proposed (De Hayr et al., 2025; Abdel-Hamid et al., 2025).

Our findings consolidate and reinforce the current understanding of *GTF3C3*-related disorder by adding a new case and integrating all known clinical data. Common clinical features include epilepsy, global developmental delay, ID and central nervous system malformations. Epilepsy commonly presents in the first years of life/early childhood, but late-onset epilepsy, such as the patient reported here, is also possible. Most frequently seizures are generalized, an observation that has important clinical implications in order to select the most appropriate treatment. In addition, our study also showed that neurological symptoms, including cerebellar symptoms, dystonic movements or spasticity, are as frequent as seizures. However, unlike seizures, these manifestations have been less emphasized in previous reports. Given their prevalence, we underscore the importance of neurological follow-up in affected individuals to better define their evolution and clinical implications.

Less frequent findings, including hearing loss and retinopathy, have been reported in a small number of previously published patients (De Hayr et al., 2025; Abdel-Hamid et al., 2025; Reuter et al., 2017). However, although no additional genetic variants or alternative etiologies were reported in the original cases, a causal relationship with *GTF3C3* cannot be established, and further studies including more patients are required to better define the spectrum of associated clinical features.

In conclusion, this work highlights the importance of regular reanalysis of unsolved cases, given the continuous discovery of novel gene-disease associations in the field of NDDs. It also supports the inclusion of *GTF3C3* in the differential diagnosis of syndromic NDDs, particularly in patients presenting with central nervous system malformations, seizures and neurological symptoms.

## Data Availability

All data generated or analyzed in this study are included in the published article. All variants are deposited in ClinVar under the following accession numbers: VCV004819552.1, VCV004819553.1, VCV004819554.1, VCV003899893.1, VCV003899892.1, VCV003899893.1, VCV003899897.1, VCV003899894.1, VCV003899896.1, VCV003233390.3, VCV003899890.1, VCV003899898.1 and VCV003899899.1. Additional information is available from the corresponding author upon reasonable request.
